# Aminoglycoside-driven biosynthesis of selenium-deficient Selenoprotein P

**DOI:** 10.1038/s41598-017-04586-9

**Published:** 2017-06-29

**Authors:** Kostja Renko, Janine Martitz, Sandra Hybsier, Bjoern Heynisch, Linn Voss, Robert A. Everley, Steven P. Gygi, Mette Stoedter, Monika Wisniewska, Josef Köhrle, Vadim N. Gladyshev, Lutz Schomburg

**Affiliations:** 1 Institut für Experimentelle Endokrinologie, Charité–Universitätsmedizin Berlin, Berlin, D-13353 Germany; 2 Department of Cell Biology, Harvard Medical School, Boston, MA 02115 USA; 3 Division of Genetics, Department of Medicine, Brigham and Women’s Hospital, Harvard Medical School, Boston, MA 02115 USA

## Abstract

Selenoprotein biosynthesis relies on the co-translational insertion of selenocysteine in response to UGA codons. Aminoglycoside antibiotics interfere with ribosomal function and may cause codon misreading. We hypothesized that biosynthesis of the selenium (Se) transporter selenoprotein P (SELENOP) is particularly sensitive to antibiotics due to its ten in frame UGA codons. As liver regulates Se metabolism, we tested the aminoglycosides G418 and gentamicin in hepatoma cell lines (HepG2, Hep3B and Hepa1-6) and in experimental mice. *In vitro*, SELENOP levels increased strongly in response to G418, whereas expression of the glutathione peroxidases GPX1 and GPX2 was marginally affected. Se content of G418-induced SELENOP was dependent on Se availability, and was completely suppressed by G418 under Se-poor conditions. Selenocysteine residues were replaced mainly by cysteine, tryptophan and arginine in a codon-specific manner. Interestingly, in young healthy mice, antibiotic treatment failed to affect Selenop biosynthesis to a detectable degree. These findings suggest that the interfering activity of aminoglycosides on selenoprotein biosynthesis can be severe, but depend on the Se status, and other parameters likely including age and general health. Focused analyses with aminoglycoside-treated patients are needed next to evaluate a possible interference of selenoprotein biosynthesis by the antibiotics and elucidate potential side effects.

## Introduction

Aminoglycoside (AG) antibiotics interact mainly with the small ribosomal subunit of prokaryotes, and to a lesser extent of eukaryotes, resulting in errors in amino acid insertion during protein biosynthesis^1^. Although AGs are rapidly excreted via the kidney, they may exert toxic side effects in patients and potentially contribute to oto- and nephrotoxicity^2^. Due to these risks, their application is limited in the clinics to local applications or serious systemic infections such as severe neonatal sepsis^3^. In veterinarian medicine, AGs are more widespread in use and even applied as a prophylactic treatment^4^.

AGs mainly affect the function of the prokaryotic 30S ribosomal subunit where they impair the proofreading activity during translation^5^. The increased error rate causes insertion of wrong amino acids into the growing peptide chain or may even result in premature termination of biosynthesis, eventually leading to non-functional proteins. Similarly, AGs also affect the eukaryotic 40S ribosomal subunit and may cause misreading of stop codons, thereby promoting extended translation products or suppressing premature stop codons (non-sense mutations). Accordingly, AG-induced stop codon read-through is explored as an adjuvant treatment option in specific inherited diseases, e.g., in the suppression of premature stop codon mutations in the CFTR or DMD gene in patients suffering from cystic fibrosis or Duchenne muscular dystrophy^6, 7^. This line of research receives increasing attention in recent years, as 10–12% of genetic diseases seem to result from in-frame nonsense mutations^8, 9^.

The opal-codon UGA has been identified as most sensitive with respect to the suppressing effects of AGs^10^. Importantly, UGA has dual functions and depending on context, it specifies stop or the insertion of the 21st proteinogenic amino acid selenocysteine (Sec) into selenoproteins. A complex biosynthesis machinery is necessary for this decoding step, involving the UGA codon, a selenoprotein-specific tRNA, the Sec-specific elongation factor eEFSEC and a specific stem loop structure within the 3′-untranslated region of the mRNA, the so-called Sec-insertion sequence (SECIS) element. The 25 genes encoding selenoproteins in humans comprise a number of well-characterized and essential enzymes, including the members of the glutathione peroxidase (GPX), thioredoxin reductase (TXNRD) and iodothyronine deiodinase (DIO) families^11^.

A seminal study has indicated that the AG geneticin (G418) interferes with GPX1 biosynthesis in COS7 cells^12^. Subsequent studies provided the first comparative data on different AGs affecting the expression of individual selenoproteins in a gene-specific manner^13^. The net effect of AGs on selenoprotein biosynthesis appears to depend on a set of parameters, including the nature of the SECIS-element, the opal-codon context (UGAN) and the actual selenium (Se) status of the cell^14^. In contrast to the other selenoprotein transcripts containing a single UGA codon, the open reading frame encoding the liver-derived Se-transporter selenoprotein P (SELENOP) contains ten UGA codons, and thus allows co-translational incorporation of up to ten Sec residues per SELENOP molecule. Moreover, the transcript harbors two separate SECIS-elements differing in their efficiency of UGA recoding^15^.

Regular SELENOP biosynthesis appears of high physiological importance for development, growth and Se metabolism, as gene knockout studies in mice indicated a number of phenotypes resulting from *Selenop* deficiency^16, 17^. In general, SELENOP is a reliable biomarker of Se status, correlating over a wide range with the Se intake in healthy humans^18^. *In vitro*, the expression of SELENOP is negatively affected by hypoxia^19^ and pro-inflammatory cytokines^20^, and an intracellular re-distribution of newly-synthesized SELENOP in response to ER stress has been reported^21, 22^. In animals and humans, it qualifies as a negative acute phase reactant^23–25^. All of these adverse conditions coincide in severe sepsis, where AGs are applied under pro-inflammatory conditions, potentially contributing to a strong and health-relevant suppression of SELENOP biosynthesis and interruption of SELENOP-dependent Se-transport to target organs. For these reasons, we decided to study its biosynthesis and sensitivity to AGs in more detail. SELENOP proved to be a most sensitive target of the disrupting effects of AGs, causing biosynthesis of Se-poor SELENOP variants in hepatocytes in culture. The nature of the Sec-replacing amino acids was not uniform but UGA codon-specific. The resulting Se-poor SELENOP variants may constitute inactive Se-transporters likely interfering with regular Se metabolism and target organ Se supply, especially in Se deficient subjects.

## Results

### Effects of aminoglycosides on hepatocyte-derived SELENOP

**Figure 1:**
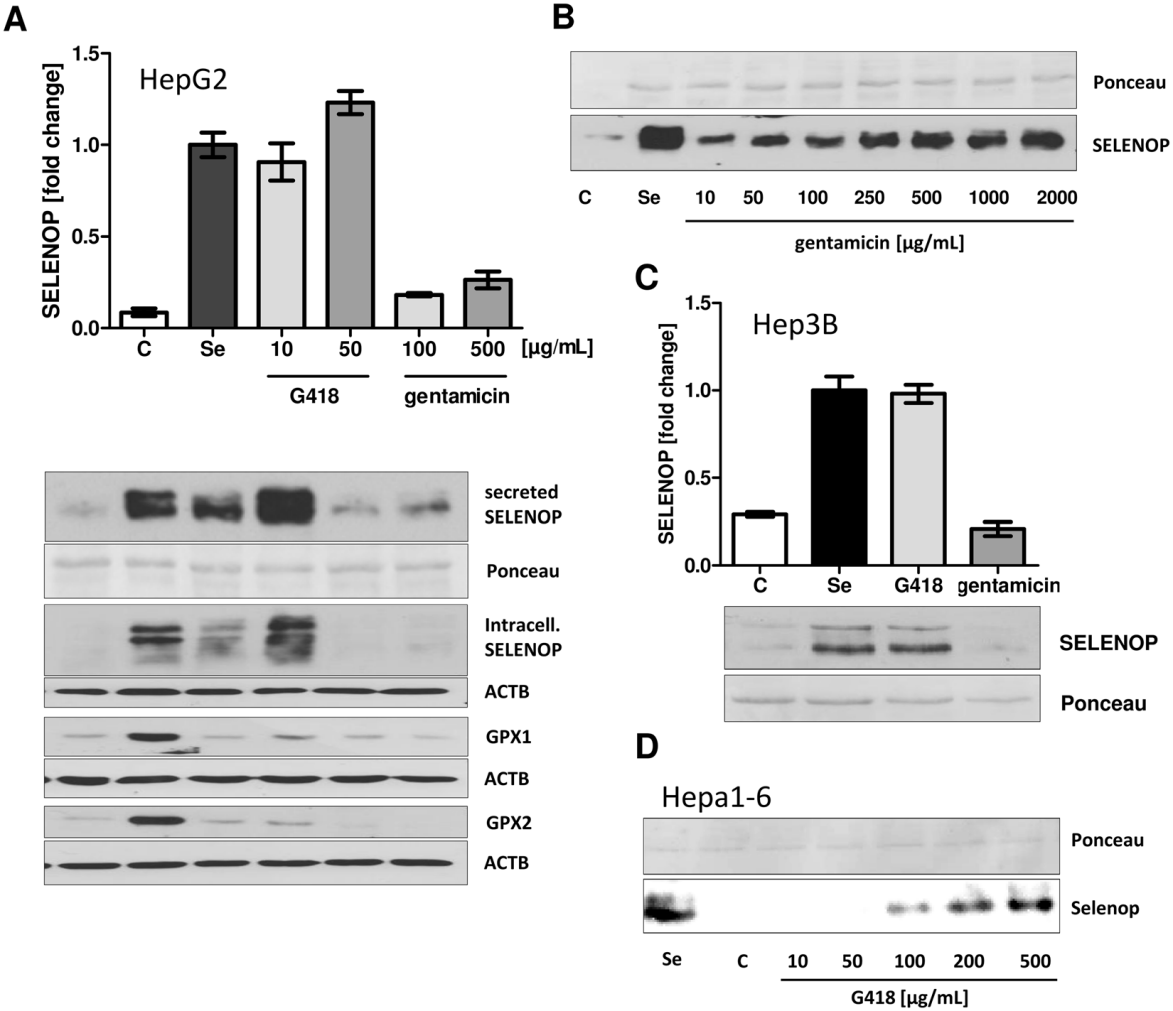
*In vitro* effects of G418 and gentamicin on selenoprotein biosynthesis. (**A**) HepG2 cells were incubated for 48 h in FCS-free medium with supplemental selenite (100 nM), G418 or gentamicin. Western blot analysis indicates increased secreted SELENOP protein levels in response to selenite and G418 treatment. Intracellular protein levels of selenoenzymes GPX1 and GPX2 were marginally affected. (**B**) The effect of gentamicin was verified in a dose-dependent analysis. (**C**) The effects of G418 on SELENOP secretion were replicated in human Hep3B, and (**D**) murine Hepa1-6 hepatocytes.

Se-depleted HepG2 cells were incubated with G418 or gentamicin; cells supplemented with 100 nM Na_2_SeO_3_ served as control (Fig. 1A). SELENOP secreted by G418-treated cells (10 or 50 µg/ml) was found in the medium in almost equal amounts as compared to that secreted by cells supplemented with selenite. In comparison, the intracellular levels of GPX1 and GPX2 were only slightly different between these conditions. The effect of gentamicin on SELENOP expression was less pronounced, but still detectable and showed a dose-dependency in HepG2 cells (Fig. 1B). The strong effect of G418 was qualitatively reproducible with human hepatoma Hep3B cells (Fig. 1C), and similarly detectable in the murine cell line Hepa1-6 (Fig. 1D). The characteristic pattern of immuno-reactive SELENOP-isoforms was unaffected by the AG-treatment as judged by Western blot analyses.

**Figure 2:**
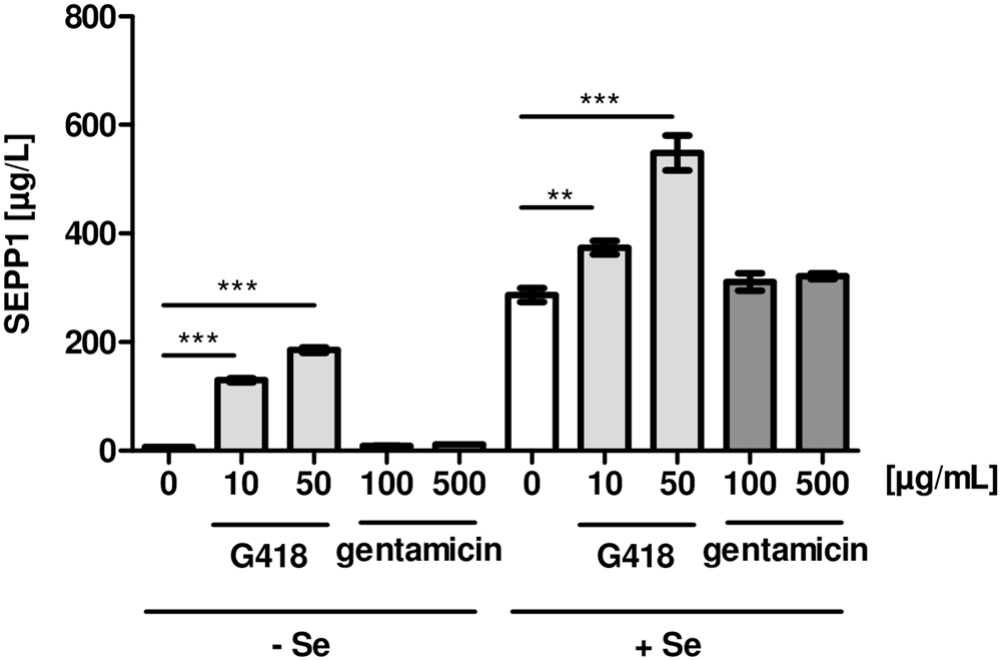
Interplay between AG and supplemental selenite on SELENOP concentrations. HepG2 cells were incubated for 48 h in the presence of G418 or gentamicin with or without supplemental selenite (100 nM). Secreted SELENOP (SEPP1) was quantified by ELISA analyses. G418 strongly induced SELENOP concentrations in the conditioned medium, whereas gentamicin showed no effect. The effect of G418 was augmented by supplemental selenite. (Mean ± SEM, n = 6, ANOVA followed by Dunnett’s).

**Figure 3:**
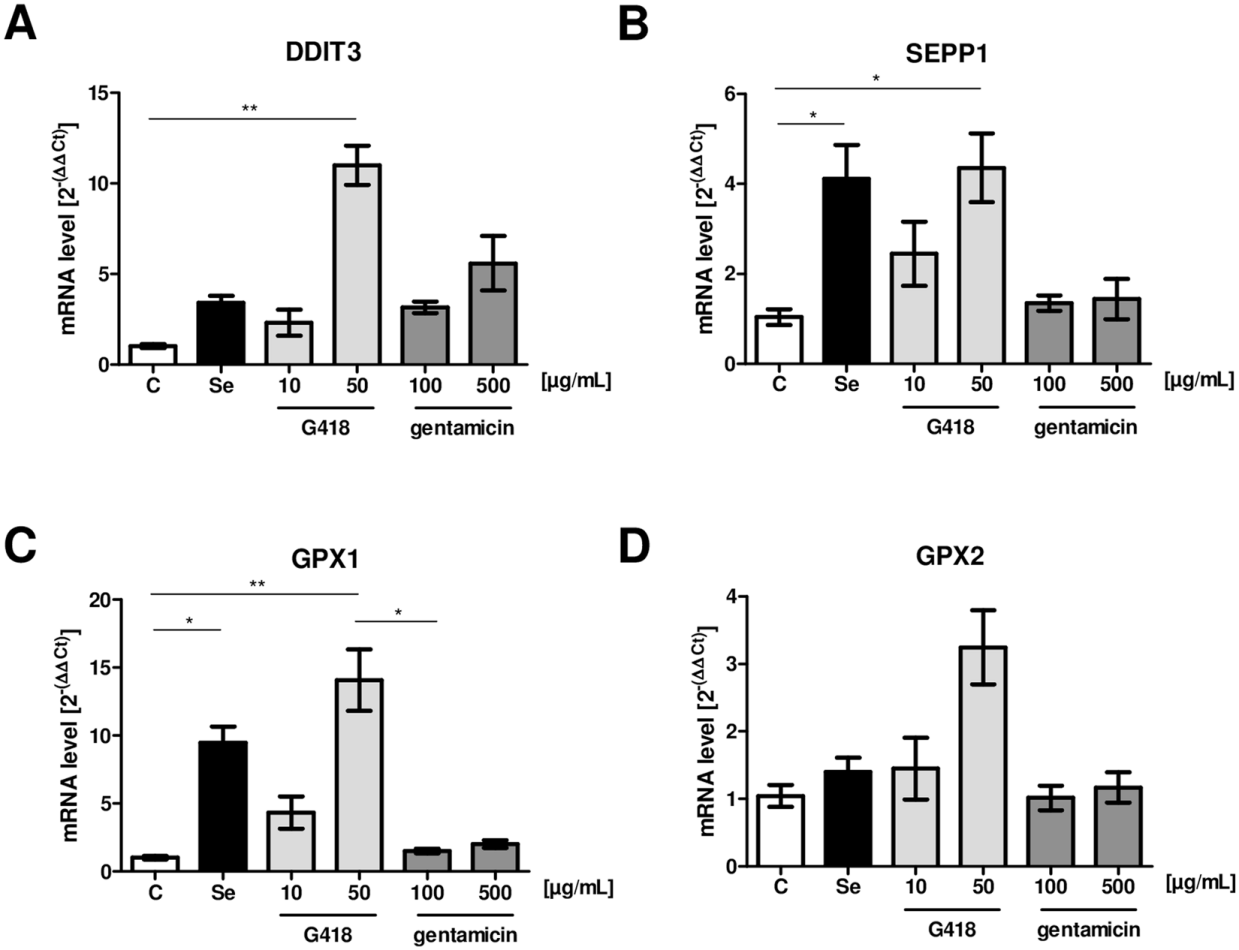
Effects of AG treatment on selenoprotein transcript levels. HepG2 cells were treated with G418, gentamicin or Se for 48 h and mRNA expression was determined by qRT-PCR using 18 S rRNA as reference. (**A**) DDIT3 served as control gene for AG effects, and transcript levels increased in response to AG treatment in a dose-dependent manner. (**B–C**) Supplemental selenite and G418 increased SELENOP (SEPP1) and GPX1 transcript levels. (**D**) Supplemental selenite or gentamicin had no effect on GPX2 transcript levels, whereas G418 showed a moderate effect. (Mean ± SEM, n = 4, ANOVA followed by Dunnett’s).

The AG-dependent increase in SELENOP expression was verified by quantitative ELISA analysis. G418-induced SELENOP production differed under Se-deficient and Se-supplemented conditions (Fig. 2). In these experiments, the effects of gentamicin were not significant while G418 effects were strong. Next, the effects of AG treatment on gene expression were analyzed. DNA damage-inducible transcript 3 (DDIT3) was chosen as positive control^26^, and increased dose-dependently in response to G418 or gentamicin treatment (Fig. 3A). Similarly, dose-dependent elevated transcript levels of GPX1, GPX2 and SELENOP were detected in response to G418 treatment (Fig. 3B–D).

### Se-content of AG-induced SELENOP

**Figure 4:**
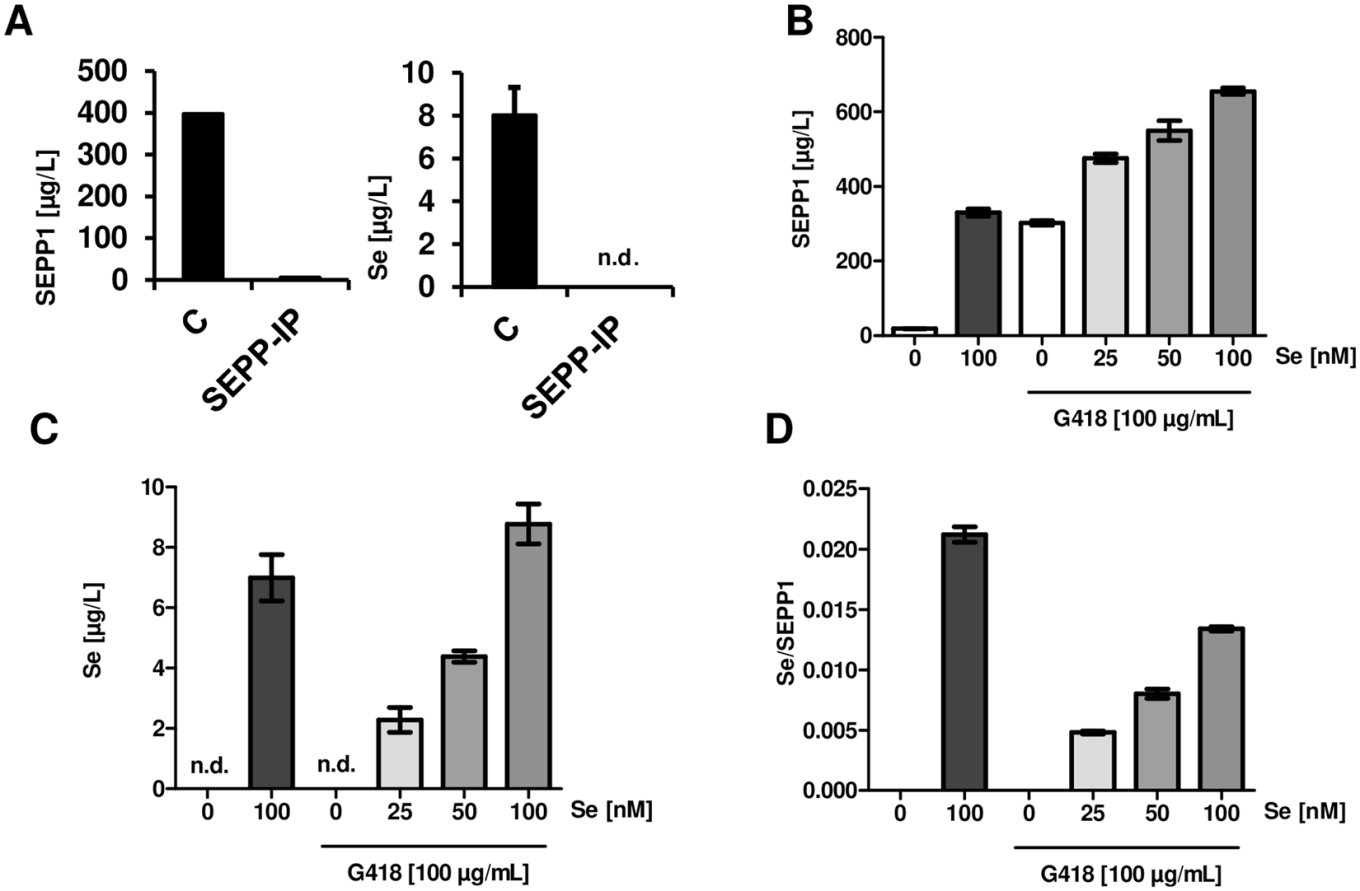
Se-content of SELENOP in relation to G418 and supplemental selenite. (**A**) The protein fraction of conditioned media from HepG2 cells was immobilized on a nitrocellulose membrane before and after immuno-precipitation of SELENOP (SEPP -IP). The SELENOP-IP procedure efficiently removed SELENOP (SEPP1), and with it all the detectable Se from the culture medium. (**B**) Supplemental selenite and G418 increased immuno-detectable SELENOP (SEPP1). (**C**) Se content of SELENOP preparations varied strongly between the different incubation conditions. (**D**) A comparison of the Se/SELENOP (Se/SEPP1) ratio indicates that G418 induces Sec-free SELENOP variants, whereas selenite increased the Se content of SELENOP in the presence of G418. (Mean ± SEM, n = 3).

As there were no indications of truncated SELENOP variants by Western blot analysis, the relative Se content of AG-induced SELENOP was determined. HepG2 cells were supplemented with 100 nM selenite and subjected to SELENOP-specific IP as positive control. The protocol efficiently removed SELENOP and all protein-associated Se from the samples (Fig. 4A). This indicates that no other major Se-containing protein besides SELENOP was present in the conditioned media. Next, HepG2 cells were treated with selenite, G418 or G418 in combination with increasing selenite concentrations. Se-content of Se- and G418-induced SELENOP differed considerably (Fig. 4B–C). Supplemental selenite induced SELENOP, and addition of G418 led to a further increase in secreted SELENOP concentrations (Fig. 4B). In the absence of supplemental Se, the G418-induced SELENOP was not containing detectable amounts of Se, i.e., G418 led to biosynthesis of Se-free SELENOP variants (Fig. 4C). Supplemental selenite increased the net biosynthesis of G418-induced SELENOP in a dose-dependent manner and increased the relative Se content per secreted SELENOP molecule gradually (Fig. 4D). These results indicate an additive effect of G418 and selenite on SELENOP biosynthesis, with selenite increasing the Se content of SELENOP, whereas G418 increased the fraction of Se-poor SELENOP variants.

### Characterization of the amino acids replacing Sec during AG treatment

**Figure 5:**
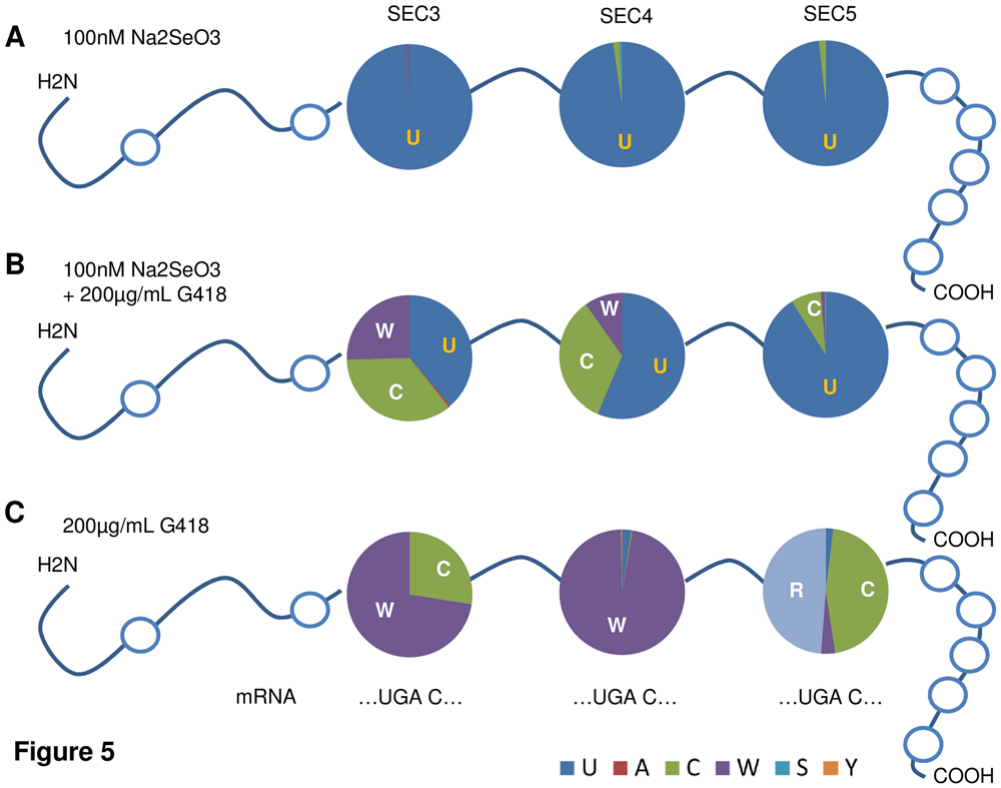
Amino acid insertion at UGA codons SEC3, SEC4 and SEC5 in SELENOP. HepG2 cells were treated with 100 nM Se, 200 µg/mL G418 or their combination. SELENOP was purified by immuno-affinity and subjected to LC-MS/MS analysis. (**A**) Sec in SELENOP was detected almost exclusively at the positions SEC3, SEC4 and SEC5 when cells were supplemented with selenite. (**B**) The pattern of amino acids inserted at the three Sec codons varied strongly when cells were grown in the presence of 100 nM selenite and 200 µg/mL G418. (**C**) SELENOP synthesized in the absence of supplemental selenite but in presence of 200 µg/mL G418 was devoid of Sec residues at the three positions available for analysis (SEC3-5). The amino acids replacing Sec were mainly tryptophan (W), cysteine (C) and arginine (R), but their relative proportions were Sec-codon specific.

In order to better characterize the molecular effects of AGs on SELENOP biosynthesis, purified protein was analyzed by LC-MS/MS. SELENOP was isolated by IP from HepG2 cells treated with 100 nM Na_2_SeO_3_, 200 µg/mL G418 or their combination. The amino acids inserted at three central UGA positions (SEC3, SEC4 and SEC5) were identified (Fig. 5). SELENOP secreted from cells supplemented with selenite carried almost exclusively the expected Sec at these positions (Fig. 5A). In contrast, supplemental G418 caused mis-incorporation at UGA codons, occasionally inserting cysteine (C) instead of the Sec (U) at position SEC5 (Fig. 5B). The ratio of incorrectly inserted residues at positions SEC3 and SEC4 was higher than at SEC5, and cysteine (C), tryptophan (W) and Sec (U) were detected at position SEC3 in almost stoichiometric amounts. The AG-induced mis-incorporation under Se-deficient conditions was almost complete at all three UGA positions. The incorrectly inserted residues showed some codon-specific features, with SEC3 and SEC4 showing a predominance of tryptophan (W), while cysteine (C) and arginine (R) dominated at the SEC5 position (Fig. 5).

### Effects of G418 and gentamicin *in vivo*

**Figure 6:**
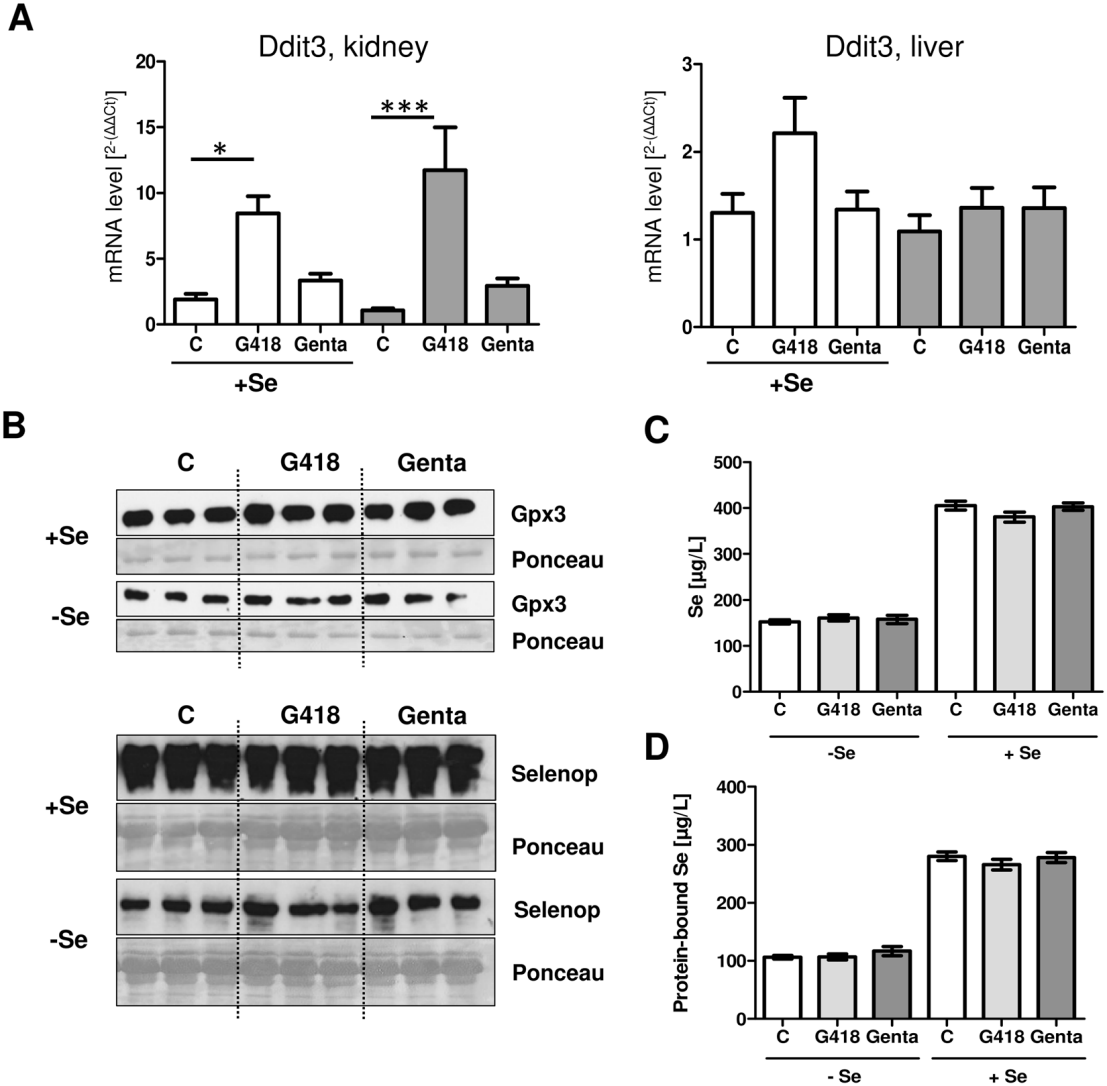
Effects of AG treatment on selenoprotein expression in mice. Mice fed Se-deficient or Se-sufficient diets were treated with G418 or gentamicin. (**A**) *Ddit3* was analyzed as an AG-responsive gene in kidney and liver, and significantly increased Ddit3 mRNA concentrations were observed in kidney upon G418 injection (ANOVA followed by Dunnett’s). (**B**) Western blot analyses of Gpx3 and Selenop showed no differences in expression levels in response to G418 or gentamicin in Se-treated or Se-deficient mice. (**C**) Se concentrations were higher in the selenite-supplemented mice than in mice raised on a Se-deficient diet. No effect of AG treatment on serum Se was detectable. (**D**) Se concentrations of precipitated protein from serum were higher in Se supplemented than in Se-deficient mice. Differences between the AG treatment groups were not detectable. (Mean ± SEM, n = 7).

Wild-type C57BL/6 J mice were chosen to test whether these rodents constitute a suitable model system for testing the consequences of the AG-induced misreading of UGA codons in selenoprotein expression in living organisms. The applicability of murine cells was supported by the results obtained before with the murine hepatocytes in culture (Fig. 1D). The animals were raised on a Se-defined diet for three weeks, then received supplemental selenite via the drinking water or not, and were injected twice with relatively high amounts of G418, gentamicin or the respective solvent, as described^27^. Efficiency of AG treatment was verified by quantifying renal Ddit3 mRNA expression, which increased strongly in response to the G418-treatment (Fig. 6A). In comparison, there were no significant effects of G418 on hepatic Ddit3 transcript concentrations. No effect of the injected AG on circulating Selenop or Gpx3 protein concentrations was evident by Western blot analyses (Fig. 6B). Serum Se concentrations were higher in the selenite-supplemented group, but no effects of the injected AG on serum Se were detectable (Fig. 6C). The same applies to the fraction of protein-bound Se in serum, which was higher in the selenite-supplemented mice than in Se-deprived mice, but remained unaffected by the AG treatment under the experimental conditions chosen (Fig. 6D).

## Discussion

The physiological transport of the essential trace element Se in the circulation depends on liver-derived SELENOP, which can be taken up by target cells in a receptor-dependent manner^18^. The use of transgenic mouse models revealed that many physiological processes, e.g. growth, immune function, brain development, male fertility, etc., depend on regular selenoprotein expression^28^. Analyses of inherited defects in humans support this notion, and show that brain, bone, the endocrine and immune responses, along with muscle and skin, are systems depending on selenoprotein function and being sensitive to impaired Se supply and disrupted selenoprotein expression^29^. For these reasons, any significant disturbance in selenoprotein expression needs to be considered as potentially harmful, no matter whether it is due to inborn errors of metabolism, nutritional deficits or side effects of pharmacological interventions.

In previous studies, AGs have been identified as potential disrupters of regular selenoprotein expression *in vitro*, based on the analyses of GPX1^12^, GPX4 or TXNRD1^13^. The consequences may be dramatic, as GPX4 and TXNRD1 are essential for life^30^. However, due to the hierarchical principles of selenoprotein biosynthesis, both enzymes become preferentially supplied with Se under conditions of Se deficiency^31^. Accordingly, they may not represent the most sensitive targets of AG-induced Sec insertion interference. In comparison, SELENOP is neither essential nor preferentially supplied with Se. Moreover, its transcript carries 10 in frame UGA codons, potentially rendering it particularly prone to AG-induced translational errors. This hypothesis was tested and verified in this study. SELENOP biosynthesis was strongly affected in all hepatic cell lines tested, and the effects were dose-dependent. The Se content of the AG-induced SELENOP variants correlated to the basal Se supply, i.e., supplemental Se was capable of competing with the disrupting effects of AG. This finding is of clinical relevance when considering adjuvant supplementation as therapeutic option for counteracting the disrupting AG effects. Surprisingly, the spectrum of amino acids replacing Sec was codon-specific. However, the strong AG-dependent effects on selenoprotein biosynthesis were not replicated in an animal model of young and healthy mice fed Se-deficient or Se-sufficient diets, indicating that this rodent model may not constitute a good choice for characterizing these interactions *in vivo*.

At first sight, it is difficult to reconcile these seemingly contradictory findings from the *in vitro* and i*n vivo* experiments. The effects in cell lines were consistent and in line with previous reports on the disrupting activities of AG on selenoprotein expression, especially with respect to G418 as a most potent aminoglycoside^12, 13^. Hepatocytes qualify as a relevant and suitable model, as the effects can reliably be monitored from the analyses of secreted SELENOP, facilitating the molecular analyses as SELENOP was abundant and constitutes the sole Se-containing protein in the conditioned media. Accordingly, a direct competition between G418 and selenite for supporting Sec-insertion into SELENOP became evident, reminiscent of the effects of thiophosphate supplementation on the balance of Cys versus Sec insertion into the selenoenzyme TXNRD1^32^. A relevance of such a competition at the UGA codons for Sec insertion in living subjects was recently demonstrated by showing that 8% of the position encoded by UGA3 in SELENOP isolated from a human plasma pool was occupied by Cys instead of Sec^33^. It is thus possible that a similar disruption of regular Sec insertion into SELENOP takes place in patients treated with high AG concentrations in the clinics, especially during applications of AG as therapeutics of severe sepsis or modulators of translational termination by supporting stop codon read-through in diseases caused by premature stop codons^6, 7^. This notion is supported by our recent analyses of the Se status in newborns with connatal infections, where we found indications that the AG treatment disrupted the linear Se to SELENOP ratio, likely due to an increased biosynthesis of Se poor SELENOP variants^34^.

Selenop biosynthesis by murine Hepa1-6 cells was also sensitive to G418, indicating commonalities of AG effects in humans and mice. This is of special interest, as the efficiency of AG-driven translational read-through depends among other factors on the base-context of UGA (especially position +4)^20^, which varies between murine and human SELENOP transcripts^35^. Gentamicin, an antibiotic with more widespread clinical use, especially in developing countries^3^, had a weaker but still detectable effect on disrupting Sec insertion into SELENOP *in vitro*. Importantly, the gentamicin used in our study was not a purified compound, but a mixtures of several related aminoglycosides. Only a minor component of gentamicin, i.e., gentamicin B1, appears to be primarily responsible for inducing translational read-through^9^. When considering a clinical application of gentamicin for treating diseases with nonsense mutations, this minor compound will likely be highly efficient at much smaller dosages than currently applied and eliciting fewer side effects. According to our results, it may be meaningful to also monitoring the Se status and avoid Se deficiency in such clinical trials, in order to minimize the risk for inducing non-functional selenoproteins that may be involved in the oto- and nephro-toxicity observed in treated neonates^2^. This risk might also be of importance in veterinary medicine, where AGs are more widely and intensively used^4^.

Besides affecting translation and amino acid insertion, AGs may affect the stability of selenoprotein transcripts by modulating the degree of nonsense-mediated decay (NMD) and/or the ribosome density downstream of UGA Sec codons, which both contribute to the hierarchical expression of essential selenoproteins relative to so-called housekeeping selenoproteins^36, 37^. And indeed, strongly elevated SELENOP and GPX1 mRNA expression was observed in response to AG treatment in hepatoma cells, likely indicating an increased translational activity successfully competing with NMD of selenoprotein transcripts^38^. As shown by Western blot analyses, AG treatment does not affect the apparent size of newly synthesized SELENOP, i.e., no premature translational termination is evident. Further analysis via LC-MS/MS revealed the postulated exchange of Sec by other residues. Interestingly, the amino acids inserted in place of Sec varied between the positions SEC3, SEC4 and SEC5, although the critical base at +4 was the same for all these Sec codons. While insertion of Cys in place of Sec might be less disrupting due to the ability of Cys to form disulfide and selenenyl-sulfide bonds, replacement of Sec with Trp or Arg is expected to lead to more severe structural changes, potentially interfering with receptor-mediated uptake.

Still, the discrepancy of the strong effects observed in hepatic cells in culture and the lack of effects in the mouse experiment is puzzling. Several factors might have contributed to these findings. a) Western blot analysis is not a very quantitative technology and small interfering effects might have gone unnoticed. b) The animals were young and healthy, and exposed to AGs for a short period of time only, which is dissimilar to the clinical situation. c) Despite being raised on a Se-deficient diet, the remaining Se, still available for selenoprotein biosynthesis, might have over-competed the interrupting effects of AGs. d) Much of the antibiotics may be excreted in live mice, in contrast to cultured cells or diseased patients, so the effects on selenoprotein expression may be less obvious. e) AG are known to accumulate more in kidney than in liver tissue, which is compatible with our findings of stronger effects on renal than hepatic Ddit3 transcript concentrations and undetectable effects on liver-derived Selenop in healthy animals. It can be speculated that any disrupting effects of AG on selenoprotein expression may become more detectable and more relevant in the clinics, where additional noxae like inflammatory cytokines^23^, hypoxia^19^ or poor nutrition and limited Se supply^36^ may synergize in impairing regular selenoprotein expression. Given that AGs are intensively used in both veterinary and human medicine, a continuation of this line of research appears promising, especially in view of the recent finding that human SELENOP contains on average only 5.4 Se atoms per SELENOP molecule in human subjects^39^.

Collectively, our data indicate strong disrupting effects of AGs on the faithful insertion of Sec residues during hepatic selenoprotein biosynthesis. Among the selenoproteins tested, SELENOP proved to be particularly sensitive. Depending on the specific UGA codon, Sec may be replaced by Cys, Trp or even Arg, likely affecting the structure and function of SELENOP. The resulting Sec-poor SELENOP variants may induce ER stress, lose their capacity of transporting Se and might even become immunogenic, promoting autoimmunity. A first evidence for SELENOP-specific autoantibodies has recently been reported, in line with previous concerns about a generally increased AG-induced autoimmunity^40^. Also in the situation of severe illness, characterized by low SELENOP concentrations, treatment with AGs may further aggravate the situation by interfering with the Se metabolism and transport. This may become of specific relevance when patients are treated with AGs over longer periods of time, e.g. in cystic fibroses, or when the organism is relatively vulnerable, e.g. adults and children in the intensive care units. Respective analyses are urgently needed, as our experiments as well as both animal and clinical studies have indicated that supplementation strategies may efficiently overcome pathophysiological symptoms resulting from insufficient selenoprotein expression.

## Material and Methods

### Cell culture

Human HepG2 and Hep3B hepatoma cells were cultured in DMEM/Ham’s F-12 (Life Technologies) medium containing 10% fetal bovine serum (FBS) in a humidified incubator at 37 °C in 5% CO_2_. Murine Hepa1-6 hepatoma cells were grown in DMEM with 10% FBS under the same conditions. To determine AG- and Se-effects, cells were grown to confluency in six-well culture dishes, starved in DMEM/Ham’s F-12 without additives overnight, washed with 1xPBS and incubated with serum-free medium and the respective supplemental AG and/or Se for an additional 48 h. Conditioned medium was removed and stored at −20 °C until analysis. Cells were washed and scraped in homogenization buffer (250 mM sucrose, 20 mM HEPES, 1 mM EDTA in H_2_O, pH 7.4) with 0.1% Triton X-100 (Merck, Darmstadt, Germany).

### Animal experimentation

Male wild-type C57BL/6 J mice were obtained from Janvier Labs (Le Genest-Saint-Isle, France). The animals received humane care in compliance with the Guide for the Care and Use of Laboratory Animals as prepared by the German National Academy of Sciences. Animal experimentation was approved by the local governmental authorities in Berlin (LAGeSo, approval no. G0064/15). The animals were raised on regular lab chow. Four weeks before treatment, all animals were adapted to a Se-deficient diet (<0.08 ppm Se, Ssniff GmbH, Germany). Starting one week before the first injection, the Se-supplemented group was supplied with additional Se via the drinking water (0.4 ppm Na_2_SeO_3_). Animals were subcutaneously injected on two consecutive days with solvent, G418 (25 mg/kg b.w.) or gentamicin (50 mg/kg b.w.), essentially as described^27^. Twenty-four h after the last injection, animals were sacrificed and tissues were prepared, frozen on dry ice and stored at −80 °C until analysis.

### LC-MS/MS analysis of SELENOP

HepG2 cells were grown to confluency in 125 cm^2^ cell culture flasks, starved for 24 h in serum-free medium and incubated for a further three days in 20 mL of serum-free medium containing the respective supplements (Na_2_SeO_3_ and/or G418). The conditioned medium was collected, pooled to a final volume of 500 mL and SELENOP was isolated by affinity purification. In brief, a SELENOP-specific monoclonal antibody (ICI-immunochemical intelligence GmbH, Berlin, Germany) was coupled to AminoLink Plus coupling gel (2 mL bed volume, Pierce Biotechnologies, Inc., Rockford, IL). Coupled antibody-loaded resin was incubated with the pooled media overnight, subsequently washed and resin-bound protein was eluted by 50 mM citric acid. Isolated SELENOP was prepared for analysis by reduction and alkylation of Cys and Sec residues with iodoacetamide. The alkylated protein samples underwent trypsin digestion and were analyzed essentially as described earlier^33^.

### Quantitative PCR

For quantitative PCR (qRT-PCR), HepG2 cells were seeded at 0.5 × 10^6^ cells per well in a six-well cell culture dish and incubated for 48 h with 100 nM Na_2_SeO_3_, G418 or gentamicin. Total RNA was extracted using peqGOLD TriFast Reagent (PEQLAB, Erlangen, Germany) and reverse transcribed using the iScript™ cDNA Synthesis Kit (BIO-RAD, Munich, Germany). QRT-PCR analyses were performed using the iCycler-System (BIO-RAD) and the ABsolute qPCR SYBR Green Fluorescein Mix (Thermo Scientific, Schwerte, Germany). Different housekeeping genes were tested for normalization and reliability under the given conditions. Finally, 18S rRNA along with the 2^−(∆∆ct)^-method was used for data evaluation. Primer sequences were as follows; human SELENOP: 5′-TATGATAGATGTGGCCGTCTTG-3′ and 5′-TGTGATGATGCTCATCATGGTA-3′; GPX1: 5′-GGGCAAGGTACTACTTATCGAG-3′ and 5′-TTCAGAATCTCTTCGTTCTTGG-3′; DDIT3: 5′-TGGGGAATGACCACTCTGTT-3′ and 5′-CTCCTGGAAATGAAGAGGAAGAA-3′ and 18S rRNA: 5′-TTGACGGAAGGGCACCACCAG-3′ and 5′-GCACCACCACCCACGGAATCG-3′, mouse Ddit3: 5′-CGAAGAGGAAGAATCAAAAACT-3′ and 5′-TCCTTCTCCTTCATGCGTTG-3′ and Actb 5′-TGCTATGTTGCTCTAGACTTCG-3′ and 5′-CACTTCATGATGGAATTGAATG-3′.

### Western Blot analysis

For Western blot analysis of conditioned medium, equal volume was mixed with 4x sample buffer (200 mM Tris-HCl, pH 7.5; 50% glycerin; 4% SDS; 0.04% bromophenol blue and 125 mM DTT). For cell lysates, protein concentrations were quantified by Pierce BCA Protein Assay Kit (Thermo Scientific) and equalized to same protein concentration. For mouse serum, samples were 10-fold diluted in 4x sample buffer. Samples were incubated for 5 min at 95 °C, cooled down and size fractionated using SDS-PAGE. Proteins were transferred to a nitrocellulose membrane by semidry blotting (BIO-RAD). Immunodetection of proteins was achieved by specific antibodies and overnight incubation: SELENOP (1:2,500 dilution, ICI, Berlin), Selenop (1:1,000 dilution, as described^41^), GPX1 (1:1,000 dilution, Abcam), GPX2 (1:5,000 dilution, kind gift of Dr. A. Kipp, DIfE, Germany) and ACTB-Peroxidase (1:25,000 dilution, Sigma-Aldrich). Quantification was performed using ECL^TM^ Western Blotting Detection Reagents (GE Healthcare, UK).

### Se-determination

For the determination of the Se-content of SELENOP, 1 mL of conditioned medium was transferred to a nitrocellulose membrane via Dot-Blotting (BIO-RAD). Defined punches (4 mm in diameter) were taken and protein was lysed by 20 µL of 60% HNO_3_ (with added Gallium (Ga)-Standard of 1000 µg/L) followed by an incubation at 70 °C for 30 min in a thermocycler. To test whether the immobilized Se originates from SELENOP, conditioned media from HepG2 cells treated with 100 nM Na_2_SeO_3_ was depleted by immuno-precipitation (IP) with a SELENOP-antibody coupled resin (1 mL resin in 5 ml conditioned medium, incubation overnight at 4 °C). Membrane–bound protein was quantified for its Se content before and after IP. To determine Se content of murine serum proteins, 40 µL of serum was diluted with water to a final volume of 200 µL and 50 µL trichloroacetic acid (100% w/v) was added. Samples were incubated on ice for 15 min, centrifuged (12,000 rpm for 10 min at 4 °C), and the resulting pellet was washed twice with ice-cold acetone. After drying at room temperature for 30 min, 40 µL of 30% HNO_3_ (containing 1000 µg/L Ga as internal standard) was added to each pellet and the samples were lysed in a thermocycler at 70 °C for 3 h. The acidic lysates were applied to total-reflection X-ray fluorescence (TXRF) sample holders, and Se was quantified via TXRF (Picofox, Bruker Nano). For serum Se analyses, serum was diluted 1:1 with the Ga-standard and measured as described above.

### SELENOP quantification by ELISA

After incubation with AGs or 100 nM Na_2_SeO_3_ and collecting the HepG2-conditioned media, or after recovery from membrane punches, SELENOP concentrations were quantified by a SELENOP-specific sandwich ELISA (Selenotest^®^, ICI, Berlin).

### Statistical analyses

Statistical analyses were performed using GraphPad Prism v4.0 (GraphPad Software Inc., San Diego, USA). Results are presented as mean + SEM. Number of replicates and tests for significance are indicated in the figure legends. The significance is assigned if P < 0.05 (*), P < 0.01 (**) or P < 0.001 (***).
